# Fever in the tropics: aetiology and case-fatality - a prospective observational study in a tertiary care hospital in South India

**DOI:** 10.1186/1471-2334-13-355

**Published:** 2013-07-30

**Authors:** Siri Kratter Abrahamsen, Cathrine Nødtvedt Haugen, Priscilla Rupali, Dilip Mathai, Nina Langeland, Geir Egil Eide, Kristine Mørch

**Affiliations:** 1 Department of Medicine, National Centre for Tropical Infectious Diseases, Haukeland University Hospital, Bergen, Norway; 2 Institute of Medicine, University of Bergen, Bergen, Norway; 3 Department of Medicine Unit 1, Christian Medical College, Vellore, Tamil Nadu, India; 4 Centre for Clinical Research, Haukeland University Hospital, Bergen, Norway; 5 Department of Global Public Health and Primary Care, University of Bergen, Bergen, Norway

**Keywords:** Fever, Aetiology, Tropics, Case-fatality, Sepsis, Malaria, Tuberculosis

## Abstract

**Background:**

The objective of this study was to describe aetiology and case fatality of fever among inpatients in a tertiary care hospital in South India.

**Methods:**

This was an observational, prospective study conducted in a tertiary care hospital in Vellore, Tamil Nadu, India. Between July 2nd 2007 and August 2nd in 2007, adult patients admitted to the hospital with temperature ≥ 38.0°C were included consecutively and followed during the hospitalisation period. Demographic and clinical data were collected and analysed for each patient. Associations were sought between death and various clinical and demographic variables.

**Results:**

One hundred patients were included, 61 male and 39 female. Mean age was 37.5 (range: 16 to 84) years. Mean fever duration was 5.4 (range: 0.1 to 42.9) weeks.

The following infectious aetiologies were recorded: tuberculosis (19%), lower respiratory infection (11%) including three with sepsis, urinary tract infection (10%) including three with *E. coli* sepsis, *Plasmodium falciparum* malaria (5%) including three patients with mixed *P. vivax* infection, scrub typhus (5%), typhoid fever (4%), cryptococcal meningitis (4%) including three HIV positive patients, endocarditis (3%) including two patients with *Staphylococcus aureus* sepsis, spleen abscess (2%), amoebic liver abscess (2%), sepsis undefined focus (1%), HIV infection (1%), hepatitis B (1%), rubella (1%), peritonitis (1%) and cholecystitis (1%).

Non-infectious causes of fever were diagnosed in 15%, including systemic lupus erythematosus in four and malignancy in six patients. Cause of fever remained unknown in 13%.

Case fatality during hospitalisation was 7% (7/100). Six of those who died were male. Five fatalities had bacterial sepsis, one spleen abscess and malignancy, and one had lymphomalignant disorder.

Diabetes and increasing age were significant risk factors for fatal outcome in unadjusted analyses, but only increasing age was a risk factor for death in adjusted analysis.

**Conclusions:**

A high number of tuberculosis and bacterial infections and a high case fatality rate from sepsis were found in this cohort, underlining the importance of microbiological diagnostics and targeted antimicrobial treatment in the management of fever. *P. falciparum* was identified in all malaria cases, and this rapidly fatal infection should be considered in patients with acute undifferentiated fever in India.

## Background

Infectious diseases are leading causes of morbidity and death in tropical countries. The World Health Organization (WHO) reports that each of the main infectious aetiologies (that is, pneumonia, diarrhoea, HIV/AIDS, malaria, tuberculosis (TB) and neonatal infections), cause between 1.05 and 0.24 million deaths respectively per year in low income countries [1].

In resource-limited settings fever may be treated empirically or self-treated due to lack of access to diagnostic tests [2]. However, clinical algorithms in order to differentiate malaria, for instance, from other causes of fever are not specific [3-5]. Knowledge of local prevalence of infections is critical in order to target clinical work up and treatment.

Nevertheless, there are only a limited number of studies from India reporting on the aetiology of fever, and reliable surveillance data are not available [6]. The main objective of this study was to describe the aetiology of fever among patients in a tertiary care hospital in South India. The secondary objectives were: a) to describe to which extent laboratory and radiological tests were used as basis for aetiological diagnoses, and b) to report on the case fatality in this cohort and investigate associations between case fatality and various demographic and clinical variables.

## Methods

### Study setting

The study was conducted in a 1750-bed tertiary care referral hospital in Vellore, Tamil Nadu, South India.

### Design

This was a prospective observational study among inpatients. Inclusion criteria were adult patients admitted to the hospital with fever of unlimited duration, with temperature ≥ 38.0°C upon admission. Co-morbidity was not an exclusion criterion. Patients were included consecutively during the four-week period from July 2nd 2007 to August 2nd 2007.

### Data collection and analysis

Data were collected from hospital records, medical staff, and patients by a researcher not involved in patient care.

Demographic data, including age and gender, were recorded prospectively, as well as clinical data including fever duration, length of stay in hospital, human immunodeficiency virus (HIV) infection, antibiotic treatment before hospital admission, history of chronic disease, symptoms and findings, treatment, final diagnoses and deaths.

Radiological, biochemical and microbiological tests performed were registered. Tests had been ordered according to the discretion of the treating physicians, and no additional tests were conducted for the purposes of the study. Blood cultures were processed through automated "BacT Alert" Biomerieux inc. Malaria was diagnosed with microscopy slides stained with Leishman stain (Fisher scientific) and with quantitative buffy coat method (QBC) (QBC Diagnostics, USA). Further were the following microbiological tests used: Scrub typhus Detect IgM ELISA (InBios International, Inc, USA), *Leptospira* IgM ELISA (Panbio Pty., Ltd., Australia), Panbio Dengue Duo IgG/IgM Cassette (Inverness, Australia), Widal Ag kit (Febrile Antigen set, Span Diagnostics Ltd., Gujarat, India), Cryptococcus antigen Latex test (Remel Inc, USA), HIV Abbot Axsym Ag/Ab combo (Abbot, Germany), Genescreen HIV Ag/Ab (BIORAD, France), HIV TRIDOT (J Mitra, India), Abbot Axsym-HBsAg ver 2 (Abbot, Germany), HBsAg EIA (Diasorin, Italy), Abbot Axsym – Anti HCV (Abbot, Germany), Ortho HCV 3.0 ELISA (Johnson and Johnson , USA).

When more than one diagnosis explaining fever had been noted in the records, the most probable aetiology (based on the available clinical information) was registered as the main aetiological diagnosis.

Data were recorded in a standardized clinical registration form.

For the patients who died, data were quality controlled from the hospital records retrospectively.

The association between death as a dependent variable and age, gender, fever duration, HIV infection and diabetes as explanatory variables were analysed by unadjusted and adjusted binary logistic regression analysis. The statistical analyses were performed using SPSS 18.0.

### Ethical considerations

The study was approved by the Regional Ethics Committee of Norway and the Institutional Research Board at the study hospital in India.

## Results

One hundred patients were included, 61 male and 39 female. Mean age was 37.4 years (range 16–84 years). Mean fever duration was 5.4 (range 0.1- 42.9) weeks. Clinical characteristics are presented in Table 1.

**Table 1:** Characteristics of 100 patients admitted with fever to a tertiary care hospital in South India during July 2007

**Characteristics**	**Patients (n = 100)**
**Age** (years)	
Mean (median, range)	37.5 (36.0, 16–84)
**Gender**	
Male (n)	61
Female (n)	39
**Fever duration** (weeks)	
Mean (median, range)	5.4 (2.1, 0.1- 42.9)
< 1 week (n)	34
1-4 weeks (n)	30
1-3 months (n)	26
> 3 months (n)	9
**Length of stay** (days)	
Mean (median, range)	11.6 (8.0, 1–57)
**Comorbidity**	
HIV^1^ (n)	6
Diabetes (n)	23

Missing values: Age, n = 2; Fever duration, n = 2.

Length of stay, n = 1; Comorbidity, missing values interpreted as no comorbidity.

^1^Six were known HIV positive; among these one had a negative

HIV test recorded, one was not tested, and four were HIV antibody positive.

Aetiological diagnoses, and associations with HIV infection and with sepsis, are shown in Table 2. Fifteen patients had more than one aetiological diagnosis.

**Table 2:** Aetiology of fever associated with HIV and with sepsis in a tertiary care hospital in South India during July 2007 (n = 100)

**Aetiology of fever**	**Patients**	**Sepsis**	**HIV positive**^**3**^
	**n = 100**	**n = 9**	**n = 6**
**Bacteria**			
Tuberculosis	19		
TB meningitis (n = 7)			
TB other (n = 12)			1
Lower respiratory infection	11	3	
Urinary tract infection	10	3^1^	
Scrub typhus	5		
Typhoid fever	4		
Endocarditis	3	2^2^	
Sepsis other	1	1	
Spleen abscess	2		
Bacterial peritonitis	1		
Cholecystitis	1		
**Parasites**			
Malaria	5		
*P. falciparum* (n = 2)			
*P. falciparum + P. vivax* (n = 3)			
Amoebic liver abscess	2		
**Fungi**			
Cryptococcal meningitis	4		2
Crypt. meningitis and TB (n = 2)			1
**Undefined**			
AUF	8		1
PUO	5		
**Viral**			
HIV	1		1
Hepatitis B	1		
Rubella	1		
**Malignancy**	6		
LMD (n = 4)			
Pulmonary (n = 1)			
Leiomyosarcoma (n = 1)			
**Non-infectious**	9		
SLE (n = 4)			
Toxic epidermal necrolysis (n = 1)			
Morbus Crohn (n = 1)			
SAPHO syndrome (n = 1)			
Arthritis (n = 1)			
Malignant neuroleptica syndrome (n = 1)			

Data are presented as number of patients (n). One patient did not receive a final diagnosis.

*Abbreviations:LMD* lymphoid malignant disorder, *TB* tuberculosis, *UTI* urinary tract infection, *HIV* human immunodeficiency virus, *AUF* acute undifferentiated fever, *PUO* pyrexia of unknown origin, *SLE* systemic lupus erythematosus; *SAPHO* synovitis/acne/pustulosis/hyperossosis/osteitis.

^1^*Escherichia coli* septicaemia.

^2^*Staphylococcus aureus* septicaemia.

^3^Six were reported as HIV positive; among these one had a negative HIV test recorded, one was not tested, and four were HIV antibody positive.

Among patients diagnosed with TB (n = 19) six patients had *Mycobacterium tuberculosis* detected in sputum samples, and for two of these it was a multi-drug resistant strain. Two patients had *M. tuberculosis* identified in lymph node biopsies. Two patients had findings consistent with TB on chest X-ray, and six on computer tomography (CT). One TB patient was also diagnosed with hepatitis C, and one with urinary tract infection (UTI). Seven patients were diagnosed with TB meningitis, five on the basis of biochemical analyses of cerebrospinal fluid (CSF). All CSF cultures were TB negative. Two patients had findings consistent with meningeal involvement on CT scans.

Bacterial infections other than TB were diagnosed in 38 patients, and pathogens identified by culture are shown in Table 3.

**Table 3:** Pathogens identified by culture among 100 patients admitted with fever to a tertiary care hospital in South India

**Pathogen**	**Blood**	**Urine**	**Bone marrow**	**Sputum**	**CSF**	**Lymph node**
	**n = 21**	**n = 24**	**n = 1**	**n = 13**	**n = 2**	**n = 2**
**Gram negative**						
*Escherichia coli*	3^1^ (14.3)	16 (66.7)				
*Klebsiella* spp*.*	1^2^ (4.8)	1 (4.2)				
*S. typhi/paratyphi*	3 (14.3)		1 (100)			
*Pseudomonas* spp.		1 (4.2)		2 (15.4)		
*Acinetobact. anitratus*				1 (7.7)		
Gram negative rods	1 (4.8)	1 (4.2)		2 (15.4)		
**Gram positive**						
*Staph. aureus*	2^3^ (9.5)					
MRSA + Enterobact.				1 (7.7)		
*Enterococcus s*pp.	1^4^ (4.8)	4 (16.7)				
*Strept. viridans*	1^5^ (4.8)	-				
Gram positive cocci		1 (4.2)				
Other	8^6^ (38.1)					
***M. tuberculosis***				6 (46.2)		2 (100)
**Fungi**						
Cryptococci	1 (4.8)				1 (50.0)	
Fungi not specified		2 (8.3)		1^2^ (7.7)	1^2^ (50.0)	

Data are given as number of positive samples with percentages in parentheses. Each patient may have had more than one positive sample.

*Abbreviations:CSF* cerebrospinal fluid, *spp* species, *S. Salmonella*, *MRSA methicillin resistant Staphylococcus aureus*, *Strept. Streptococcus*, *Staph Staphylococcus*, *Enterobact*. *Enterobacteriaceae*, *M mycobacterium*.

^1^Samples from three patients with urosepsis.

^2^Sepsis with undefined focus.

^3^Samples from two patients with endocarditis.

^4^Spleen abscess.

^5^Pulmonary infection and sepsis.

^6^Spores, 3; difteroides, 2; gram positive bacteria and difteroides, 1; micrococci, 1, staphylococcus spp, 1.

Three out of 11 patients diagnosed with lower respiratory infection (LRI) died of sepsis. Diagnosis was confirmed in six patients by chest X-ray and in two by CT. One patient had Gram-negative rods in sputum, two patients had lung abscess.

Among 10 patients diagnosed with UTI, based on urine microscopy and culture, three had *E. coli* sepsis. Nosocomial UTI was found in 10 patients with other main causes of fever.

*Plasmodium falciparum* malaria was found in 5 patients based on Quantitative Buffy Coat (QBC) and thin and thick blood smears. Among these, three patients were diagnosed with mixed *P. vivax* infection. Parasitaemia in the range between 5% and 15% was recorded in three patients.

Scrub typhus (n = 5) was diagnosed based on positive Weil Felix and positive IgM ELISA in one patient, while four were diagnosed based on symptoms and findings (headache, myalgia, lymphadenopathy and eschar). One of these patients also had positive HBsAg.

All patients diagnosed with cryptococcal meningitis (n = 4) had cryptococcal Ag detected in CSF. One had disseminated infection with cryptococci isolated from blood, urine and CSF cultures. CT was negative in all four patients. Three of these patients had known untreated HIV, and among these one also had TB. One was HIV negative and had TB.

Typhoid fever (n = 4) was diagnosed based on positive typhi-dot rapid test (n = 3), Widal (n = 2) and culture (n = 4). All patients had *Salmonella typhi* (n = 3) or Gram-negative rods (n = 1) in blood culture. One patient had Gram-negative rods in blood culture and *S. typhi* in bone marrow biopsy.

Among patients with endocarditis (n = 3), two had *Staphylococcus aureus* in blood culture and one of these patients died. One patient was culture negative. Two had rheumatic heart disease.

Spleen abscess (n = 2) was diagnosed based on clinical and radiological findings, and one of these died; one had *Enterococcus* spp. in blood culture.

Among patients diagnosed with sepsis (n = 9), five patients died. Microbiological and clinical information among these are shown in Tables 3 and 4 respectively.

**Table 4:** Characteristics, diagnoses, microbiological findings and treatment among patients who died during hospitalisation in a cohort of 100 patients admitted with fever to a tertiary care hospital in South India (n = 7)

**P**	**Fever (days)**	**Final diagnosis**	**Microbiological findings**	**Anti-microbial treatment**^**1**^	**LOS**
					**(days)**
1	105	Spleen abscess	Blood culture: Diphteroides^3^	Meropenem	22
2	90	LMD^2^	Sputum*: Acinetobacter anitratus*	Metronidazol, ampicillin, cloxacillin, gentamycin	20
			Urine: *E-coli*		
3	5	Sepsis	Sputum: MRSA, enterobacteriaceae	Imipenem, penicillin, ciprofloxacin, metronidazol	14
			*Pseudomonas spp*		
			Blood: *Klebsiella*		
4	15	Pulmonary infection and sepsis	None	Ampicillin	3
5	30	Endocarditis and sepsis	Blood: *Staphylococcus aureus*	Penicillin G, cloxacillin	1
6	3	Pulmonary infection and sepsis	Sputum and CSF: Fungus	Penicillin G	16
7	3	Pulmonary infection and sepsis	Blood: *Streptococcus viridans*	Penicillin G, doxycyclin, cefotaxime	7

Abbreviations: *P* patient, *Neg.* negative, *LMD* lymphomalignant disorder, *UTI* urinary tract infection, *MRSA* methicillin resistant *Staphylococcus aureus*, *LOS* length of stay, *SLE* systemic lupus erythematosus.

^1^Antibiotics given as combination treatment or successively.

^2^Hairy cell leukaemia found by bone marrow histology.

^3^Probably due to contamination of sample from skin.

Amoebic liver abscess (n = 2) was diagnosed based on clinical findings and abdominal ultrasound.

Five among seven patients with malignancy had lymphoid malignant disorder (LMD) based on biopsy from bone marrow, skin or lymph node, and among these one patient died who had spleen abscess as an infectious aetiological diagnosis of fever. One had adenocarcinoma in lung and brain, and one had leiomysarcoma. Systemic lupus erythematosus (n = 4) was diagnosed based on dsDNA, anti-nuclear antibodies and biochemical tests. Five patients had other non-infectious aetiological diagnoses as shown in Table 2. In 13% of patients a microbiological aetiology of fever was not identified; among these, those with fever duration less than three weeks were diagnosed as acute undifferentiated fever (AUF) and those with fever more than three weeks as pyrexia of unknown origin (PUO).

Diagnostic yields among radiological and microbiological tests performed in this cohort are shown in Table 5. Chest X-ray was performed in the majority of patients, and had a diagnostic yield of 8%, while MRI was performed in 5 patients with a diagnostic yield of zero. HBsAg and hepatitis C antibodies were analysed in approximately two thirds of all patients, with a diagnostic yield of less than 3%. Scrub typhus IgM ELISA was used as a diagnostic test in 20 patients; among these were all patients with AUF and PUO, with a diagnostic yield of 5%. All positive malaria QBC tests (n = 5/77) were also analysed by microscopy, which then had a diagnostic yield of 100%. Dengue IgM ELISA was used as a diagnostic test for four patients; one test result was positive but the patient was for unknown reasons not diagnosed with dengue fever.

**Table 5:** Use of diagnostic procedures among 100 patients admitted with fever to a tertiary care hospital in South India

**Diagnostic procedure**	**Patients tested**	**Diagnostic yield**^**1**^	
	**n**	**n**	**(%)**
Chest X ray	92	13 /166	(7.8)^2^
CT	31	16 /31	(51.6)
MRI	5	0 /5	(0.0)
Abdominal US	60	13 /60	(21.6)
HBsAg detection	69	2 /69	(2.9)
Anti-HCV detection	64	1 /64	(1.6)
Anti-HIV detection	72	4 /72	(5.6)
Scrub typhus IgM ELISA	20	1 /20	(5.0)^3^
Weil felix	7	1 /7	(14.3)
Typhi dot	36	3 /36	(8.3)
Widal	14	3 /14	(21.4)
Leptospira IgM ELISA	15	0 /15	(0.0)^4^
Dengue IgM ELISA	4	1 /4	(25.0)
QBC	77	5 /134	(3.7)
Malaria microscopy	5	5 /5	(100)
Stool microscopy	17	1 /17	(5.9)
Blood culture, automated	83	11 /157	(7.0)^5^
Urine culture	42	21 /42	(50.0)
CSF culture	16	1 /16	(6.3)
Sputum culture	53	10 /53	(1.9)

Abbreviations: *CT* computer tomography, *MRI* magnetic resonance imaging, *US* ultrasound, *HCV* hepatitis C virus, *HIV* human immunodeficiency virus, *CSF* cerebrospinal fluid, *HBsAg* Hepatitis B surface antigen, *QBC* quantitative buffy coat.

^1^Positive tests among total number of tests performed.

^2^74 chest X-rays were not described at discharge.

^3^Results were not available for nine patients at discharge.

^4^Results were not available for six patients at discharge.

^5^Results from 46 blood cultures were not available at discharge.

Results were not available from 45% of chest x-rays and 29% of blood cultures at the time of discharge. Serology results for scrub typhus and leptospirosis were not available for 45% and 40% of tests performed, respectively.

Case fatality in this study was 7% (7/100). Characteristics, findings and treatment among patients who died in hospital are shown in Table 4. Two patients (with the diagnoses disseminated cryptococcosis and staphylococcal endocarditis) were discharged after only a brief hospital stay due to short expected lifespan, indicating that the case fatality rate of 7% is an underestimate in this cohort.

In unadjusted analysis, diabetes and increasing age were significant risk factors for a fatal outcome, while only age was found to be a risk factor for death in adjusted analysis (Table 6). Among men, 10% died compared with 3% of women, but this difference was not statistically significant. None of the HIV positive patients died during hospitalisation.

**Table 6:** Logistic regression of deaths in a cohort of 100 patients admitted with fever in a tertiary hospital in South India associated with characteristics and comorbidity

**Variables**	**N**	**Deaths (n = 7)**										
		**Yes**	**No**	**Unadjusted**^**a**^			**Fully adjusted**^**b**^			**Final model**^**c**^		
		**n (%)**	**n (%)**	**OR**	**95% CI**	**p-value**	**OR**	**95% CI**	**p-value**	**OR**	**95% CI**	**p-value**
**Gender**												
Women	39	1 (2.6)	38(97.4)	Ref.			Ref.					
Men	60	6(10.0)	54(90.0)	4.22	(0.49,36.51)	0.191	4.72	(0.44,51.16)	0.202			
**Age**												
per 10 years^d^	98	56.7	36.0	2.46	(1.38,4.37)	0.002	2.42	(1.20, 4.91)	0.014	2.44	(1.37,4.34)	0.002
**Fever duration**^d^	98	5.4	5.1	1.00	(0.90,1.10)	0.930	0.97	(0.83, 1.12)	0.692			
**Comorbidity**												
Diabetes	24	4(16.7)	19(79.2)	5.20	(1.07,25.20)	0.041	1.15	(0.14, 9.38)	0.898			
HIV	6	0 (0.0)	6 (100)	.00	0.00	0.999	0.00	.00	0.999			

*Abbreviations:OR* odds ratio, *CI* confidence interval, *HIV* human immune deficiency virus.

^a^Simple binary logistic regression analyses

^b^Multiple binary logistic regression analysis including all variables in one model.

^c^Backward stepwise elimination of variables with likelihood ratio test p-value ≤ 0.05 starting from fully adjusted model.

^d^Data are given as mean age (years) and mean fever duration (weeks).

## Discussion

In this study among a cohort of patients admitted to a tertiary care hospital in South India with fever, bacterial infections (38%) and TB (19%) were the most common aetiological diagnoses. Mean fever duration was high (5.4 weeks), and therefore the relatively high prevalence of TB is not surprising and is consistent with other hospital based studies reporting aetiology of prolonged fever in India. One study among 100 patients with fever of unknown origin (PUO) in Calcutta reported that TB (53%), neoplasms (17%) and collagen vascular disorders (11%) were the dominant causes [7]. A similar pattern was found in two studies of PUO in North India, where infectious causes were diagnosed in approximately half of all cases, and TB was the most common infectious aetiology [8,9]. In a study of PUO from the South of Turkey, the most important infectious causes (59%) included TB (17%), endocarditis (7%), intra-abdominal abscess (7%), brucellosis (6%), and UTI (6%) [10].

Bacterial infections and sepsis were associated with all the deaths recorded in this cohort, except for one patient who died due to malignancy. Not surprisingly among patients with severe bacterial infections, older age was a significant risk factor for death. In general, bacterial sepsis is probably undertreated in tropical countries for several reasons, including unspecific diagnosis of fever, high prevalence of multi-resistant microbes, lack of blood culture facilities, and widespread use of counterfeit drugs. This may explain the high case fatality reported in African patients with fever incorrectly treated as malaria [11]. In India, a retrospective study from Mumbai among 160 patients who died from acute febrile illness reported malaria in 23%, leptospirosis in 22% and dengue fever in 2%, while up to 54% died due to unexplained fever, a proportion of which is likely to have been caused by bacterial infections [12]. However, in our study, as many as 83 among 100 patients were tested with blood culture, and all patients with AUF and PUO had negative blood cultures (except one PUO who was not tested), suggesting that bacteraemia may not have been under-diagnosed in this cohort. Antimicrobial resistance patterns were not recorded in all patients in this study, but it is possible that antimicrobial resistance has had an impact on case fatality. Antimicrobial resistance is a well-described predictor of increased fatality rate in sepsis [13].

Malaria is known to be severely underreported and insufficiently controlled in India [6,14], and malaria overtreatment, at the cost of other potentially severe infections, has also been reported [15]. Malaria prevalence in South India has been reported to be low compared with states in North-East India [16]. Among hospitalised cases in Vellore, Tamil Nadu in 1998, a predominance of *P. vivax* infections (n = 97) compared with *P. falciparum* (n = 70) was reported [17]. In our study 5% of fever cases were due to malaria, all of which involved *P. falciparum* infections. A malaria prevalence of 17% among patients hospitalised with acute undifferentiated fever was reported from Vellore, India in 2010, and, among these, 57% were due to *P. falciparum*[18]. A similar increasing trend of *P. falciparum* predominance has been reported from Central India [19,20].

The ratio of infectious causes of fever compared with non-infectious causes is usually higher among immunocompromised patients than immunocompetent individuals, as previously reported in two studies from hospitalized patients in India and Thailand [21,22]. In these studies, fever in HIV patients was caused by infections in 100% and 85% of patients respectively, with TB (69% and 42%), cryptococcal infections (10% and 24%) and *Pneumocystis jiroveci* pneumonia (7% and 13%) as the most common causes [21,22]. In our study, six patients had HIV infection, and three of these presented with cryptococcal infection.

Scrub typhus and dengue virus infection, which typically cause acute fever, were diagnosed in 5% and 0% of the patients respectively. These figures are low compared with 48% and 7%, respectively, reported from a prospective study among patients from the same hospital in 2010 [18]. This difference may be explained by selection criteria or other study variables, including seasonal variations or the occurrence of epidemics. Our study was conducted during the hot dry season (July), while Chrispal et al. included patients for 12 consecutive months. Scrub typhus is more prevalent during cooler months, and outbreaks of dengue are reported in the area [23-26], but the difference may also be due to undetected cases. In our study cohort, only four cases were tested for dengue infection, and only nine out of 20 results of tests for Scrub typhus were available at the time of discharge.

The prevalence of leptospirosis in the area is known to be high; Chrispal et al. detected 12 cases of leptospirosis among hospitalised cases of fever (3%) in 2010, and a seroprevalence of approximately 70% has been reported in exposed risk groups, such as rice mill farmers, in the area [27]. Leptospirosis may potentially have been under diagnosed in our cohort, since only nine serological tests were available at the time of discharge.Hantavirus infection in humans in India was first reported from Vellore [28], and is most probably under diagnosed in India, as reported by Chandy et al. [29]. Hantavirus was found in one case among 398 hospitalised AUF patients in Vellore by Chrispal et al. [18]. Hantavirus serology was not performed in patients in our cohort. Furthermore, *Brucella* serology was not performed, and since blood cultures need three weeks incubation to detect *Brucella* spp., this is another example of an infection that potentially may have been overlooked.

The aetiology of fever among travellers to the tropics differs from that among residents due to pattern of exposure and immunity, as was shown in a study among 1743 western travellers with fever imported from the tropics in Belgium, where TB was diagnosed in only 2% [30]. In this large study, travellers to Asia were diagnosed with LRI (13%), dengue fever (12%), non-falciparum malaria (9%), bacterial enteritis (9%), mononucleosis-like syndrome (7%), skin/soft tissue infection (4%), enteric fever (3%), genitourinary infection (3%) and *P. falciparum* malaria (2%), while 19% had fever of unknown aetiology [30]. A study among travellers from Asia in Australia reported a similar pattern with bacterial pneumonia, dengue fever, malaria, hepatitis A and typhoid fever reported as relatively common infections, while TB was diagnosed in only 0.4% [31].

The pattern of fever aetiology found in this study would not be representative for the causes of fever in the general population. There are three main limitations explaining this. The selection of patients admitted to a tertiary care hospital in India is biased as a result of factors such as severity of disease, gender and accessibility. In this study only adults where included, while children would expect to have different fever aetiologies due to exposure and immunity. Entomological factors cause seasonal and geographical variations in vector borne diseases in India, and our study selecting patients during a dry month of the year in South India would not incorporate such variations.

Further studies are needed, both population- and hospital based, in order to provide more evidence based information about the prevalence of infectious diseases in India.

## Conclusion

A high number of bacterial and TB infections were found in a cohort of adult patients admitted to a tertiary care hospital with fever in South India, and there was a relatively high case fatality rate from sepsis. This underlines the importance of establishment of relevant microbiological diagnostics and targeted antimicrobial treatment in the management of fever. *P. falciparum* was identified in all malaria cases, and this rapidly fatal infection should be considered in all patients with acute undifferentiated fever in India.

## Consent

Written informed consent was obtained from the patients for the publication of this report.

## Competing interests

The authors declare that they have no competing interests.

## Authors’ contributions

SKA, CNH, KM, NL and DM designed the study. Collection of prospective data was performed by SKA and CNH, supervised by DM and PR. Statistical analyses were performed by GEE and KM. All authors contributed to interpretation of results and revision of the manuscript. All authors read and approved the final manuscript.

## Pre-publication history

The pre-publication history for this paper can be accessed here:

http://www.biomedcentral.com/1471-2334/13/355/prepub
